# UK haemophilia consultant access to foot and ankle services and concurrent patient impact questionnaire responses to foot and ankle interventions

**DOI:** 10.1111/hae.14625

**Published:** 2022-07-13

**Authors:** Richard A. Wilkins, Heidi J. Siddle, Graham J. Chapman, Elizabeth Horn, Rebecca Walwyn, Anthony C. Redmond

**Affiliations:** ^1^ Leeds Institute of Rheumatic and Musculoskeletal Medicine (LIRMM) University of Leeds Leeds UK; ^2^ Leeds Haemophilia Comprehensive Care Centre Leeds Teaching Hospitals NHS Trust Leeds UK; ^3^ School of Sport and Health Sciences University of Central Lancashire Preston UK; ^4^ Clinical Trials Research Unit, Leeds Institute of Clinical Trials Research University of Leeds Leeds UK; ^5^ NIHR Leeds Biomedical Research Centre Leeds Teaching Hospitals NHS Trust Leeds UK

Dear Editor,

Multi‐joint haemarthropathy is a common feature of severe haemophilia. More recent focus has been placed on moderate haemophilia where patients who experience haemarthrosis are similarly affected, requiring the initiation of prophylaxis treatment regimens.^1^ Although, the prevalence and incidence of haemarthrosis affecting the elbows, knees and ankles are similar in prophylaxis compliant patients with severe non‐inhibitor haemophilia, the ankle is disproportionately affected by haemarthropathy.^2^ The burden of ankle joint disease is great with high levels of patient‐reported pain and disability. Improvements in health‐related quality of life (HRQoL), reduction in bleed rates and improvement in pain are reported when footwear and foot orthoses are provided, however, there is yet to be any national guidance on use.^3^, ^4^

United Kingdom Haemophilia Centre Doctors Organisation (UKHCDO) quality standards of care for people with haemophilia and other bleeding disorders recommend timely access to clinical services related to the management of musculoskeletal (MSK) disease including orthopaedics, rheumatology, specialist physiotherapy, psychology and orthotic services.^5^ The Quality review report 2020 of Inherited and Acquired Haemophilia and other Bleeding Disorders (IABD) identified the under‐provision or complete absence of core and extended multi‐professional team members as the most frequent and significant concern reported.^6^ Understanding the clinical services available to haemophilia consultants and patients will provide a snapshot of current UK National Health Service (NHS) clinical services available to manage ankle haemarthrosis and haemarthropathy.

To obtain insight, patients identified from a multi‐centre HRQoL study of patients with severe and moderate haemophilia and ankle haemarthropathy (n = 241) were asked to provide details of clinical specialist services attended of MSK orthotic services, foot and ankle assessment and foot orthoses provision (IRAS:206141, REC:16/LO/2251). Patients were asked if they had access to a podiatrist/chiropodist for nail cutting and callus removal. Details of hospital‐provided or adapted shoes, the provision of foot orthoses and point of access (shop brought, NHS or private provision) were recorded.

A simultaneous survey of haemophilia consultants was undertaken (Online surveys, Bristol, UK, Jisc 2021) and distributed to all UKHCDO registered consultants; consent was provided through the submission of the online survey. Consultants were asked to provide details of services offered at their respective haemophilia centres related to the management of all MSK disease and UKHCDO care standards.^6^ They were asked to provide specific details relating to the management of the foot and ankle by orthotic services, MSK assessments and the provision of foot orthoses provided by podiatry/chiropody services, as well as access to nail and skin (corns and callus) care. The following aspects of care were also assessed; access to foot and ankle orthopaedic services, rheumatology, psychology, radioactive synovectomy (RS), point of care ultrasound (POCUS) and physiotherapy services.

A total of 241 patients with severe (n = 212) and moderate haemophilia (n = 29), with ankle haemarthropathy were collected. The mean (SD) age of patients were 43.1 (13.0) and 48.4 (SD 15.9) for severe and moderate haemophilia, respectively. Patients provided details of accessing services for foot and ankle care; physiotherapist, podiatrist/chiropodist for musculoskeletal assessment, footwear and foot orthoses provision. Overall, 41 consultant survey responses were received from 28 comprehensive care centre (CCC) and 13 haemophilia treatment centres (HC) across the UK.

Concerning access to clinical care, both consultant survey and patient questionnaire responses are compared in Figures 1 and 2. Consultant‐reported access to specialist services (Figure 1) at haemophilia centres was indicated either directly or indirectly in all settings for rheumatology and orthopaedics. POCUS was minimally available, with a total of 19 (43%) centres reporting no access (11 CCC, 8 HC). Consultants at seven (17%) centres had no access to RS services (3 CCC, 4 HC) and four (9%) centres (3 CCC, 1 HC) had no access to psychology. A total of 13 (32%) centres (8 CCC, 3 HC) had no access to two services (POCUS, RS, physiology, routine podiatry), with only one (2%) HC without access to three clinical services (routine podiatry, POCUS, RS). Orthopaedic, rheumatology, physiotherapy, podiatry and orthotic services were available by direct or indirect referral within haemophilia centres nationally.

**FIGURE 1:**
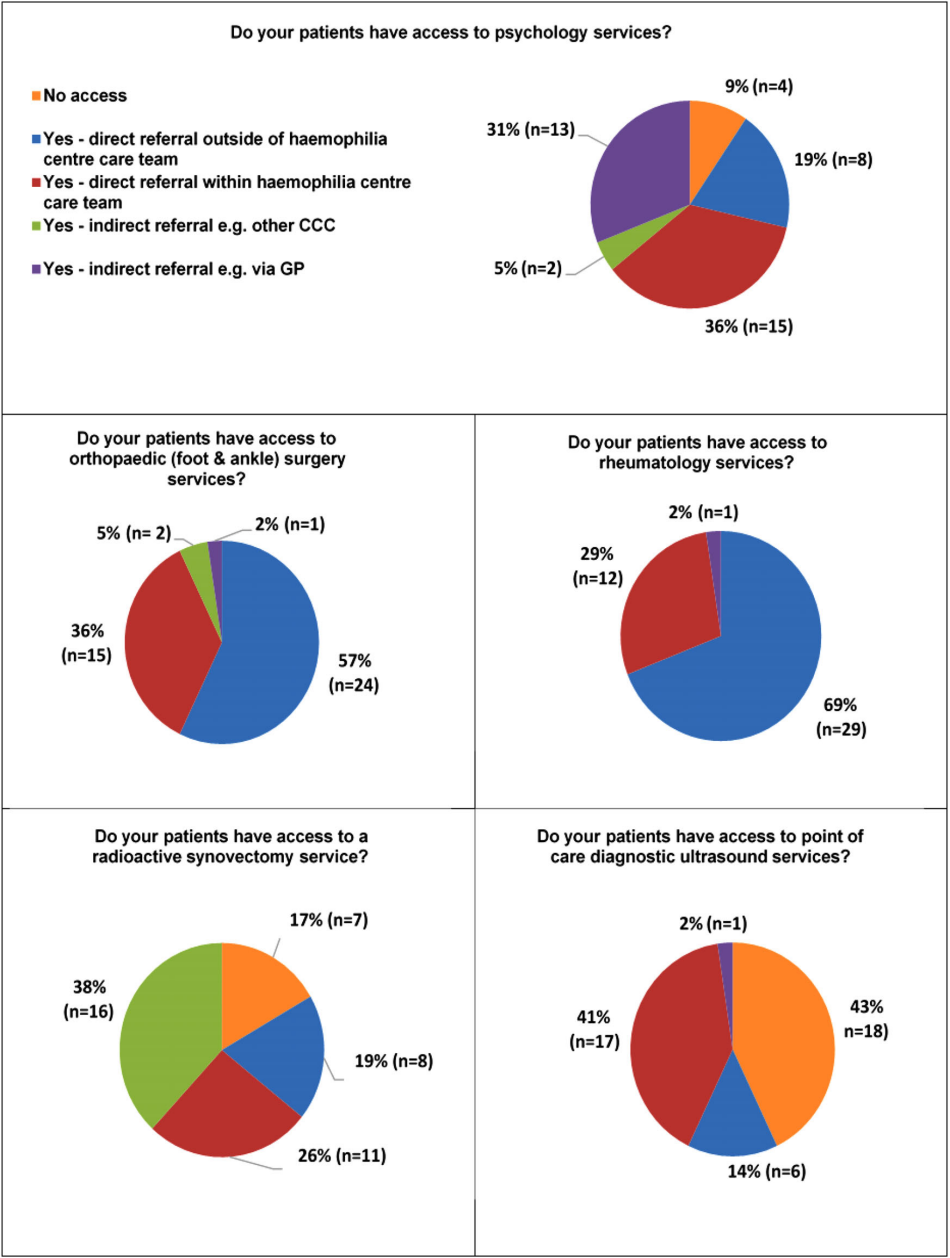
Consultant access to specialist services

**FIGURE 2:**
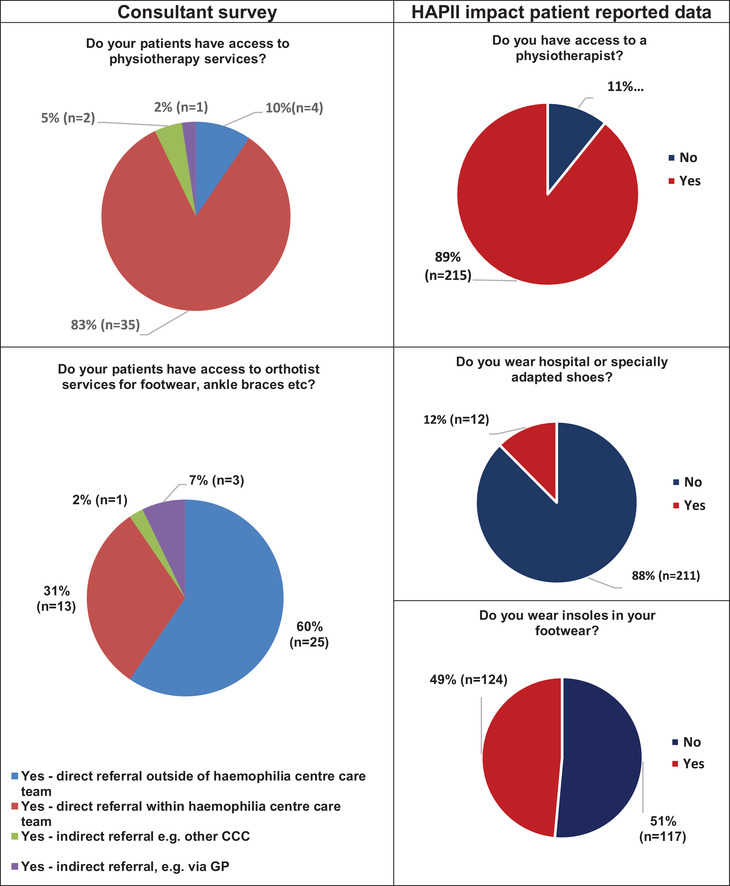
Consultant and patient reported access to podiatry musculoskeletal services

Access to physiotherapy and foot and ankle services are presented in Figure 2. In the consultant survey, all (n = 42, 100%) haemophilia centres reported direct or indirect access to physiotherapy and orthotic services. In comparison 26 (11%) patients reported no access to a physiotherapist.

Finally, consultants were asked whether they had access to a podiatrist at their corresponding CCC or HC, to which all centres had direct or indirect access. In contrast, only 101 (42%) patients reported having access to a podiatrist; 81 (34%) were given foot orthoses. For the patients that used foot orthoses, 73 (63%) were supplied by the NHS, 36 (31%) were shop brought and 7 (6%) obtained foot orthoses from a private podiatrist. Consultants indicated services for nail care, corn and callus management were available in 38 (90%) centres. In contrast, only 20 (8%) patients reported access to routine treatment (nails, corns callus) by a podiatrist, with 133 (57%) indicating this did not apply to them.

A UK survey of haemophilia consultants and data from the concurrent patient questionnaire has identified that patients with haemophilia and ankle haemarthropathy have direct or indirect access to a range of services to manage their foot and ankle complications. Only a small number of centres were unable to access specific services such as POCUS, RS and psychology services. These findings are comparable to the data published by the Care Quality Review of IABD programme 2019/2020 on behalf of the UKHCDO.^6^ Despite improvements in access to physiotherapy, there are still centres across the UK that do not have access to a specialised haemophilia physiotherapist and therefore patients may be missing a key aspect of their MSK care.^7^ Physical therapy in haemophilia has been shown to reduce pain and provide expertise in the management of bleeding disorders by limiting joint damage, improving patient pain and disability.^7^ POCUS is an emerging imaging modality in haemophilia used in the assessment of joint pathology. POCUS is a low‐cost and reliable method of assessing joint status such as effusion and synovial hypertrophy, however, 43% (n = 18) of centres in the consultant survey did not have access. Use of POCUS may improve patient outcomes as well as decrease the impact of disease by timely assessment of joint swelling, pain and monitoring joint health, however, POCUS is yet to be fully utilised in UK haemophilia care.^8^

The finding of this consultant survey and patient questionnaire suggest that whilst access to routine foot care (nail and skincare) is available, a large proportion of participants did not deem it applicable with only 20 (8%) using the services. There is no evidence to support the need for regular foot care in haemophilia, however, increased plantarflexion deformity combined with axial deformity may limit the patient's ability to self‐care. This is a common feature of rheumatoid arthritis where the loss of hand strength and foot deformity limits self‐care and foot deformity leads to the build‐up of painful callosities.^9^ The finding of this survey and patient questionnaire suggests the level of disability may not limit self‐care, or patients rely on family members to provide foot care.

Access to footwear and orthotics was available at all centres in the consultant survey however, 211 (88%) patients in the patient questionnaire reported not using adapted shoes and only 117 (51%) used foot orthoses. Differences were reported with access to podiatry services, potentially limiting the number of patients who are supplied with footwear and foot orthoses. The efficacy of footwear and foot orthoses in a Belgian cohort of haemophilia patients report good patient satisfaction with 63% (n = 10) of patients stating significant reductions in pain and improvement in comfort.^10^ In the UK only two centres have combined physiotherapy and podiatry services that provide footwear and foot orthoses, both of which report reductions in pain, improvement in HRQoL and high levels of patient satisfaction.^3^, ^4^ Use of modified footwear was limited to 12 (12%) patients in the patient questionnaire and 51% (n = 117) did not use of foot orthoses. In the majority of cases, foot orthoses were obtained from NHS clinical services, but 36 (31%) patients used shop‐bought orthoses. For these patients access to orthotic or podiatry services may not be available, or previous intervention resulted in poor satisfaction. In clinics, this is often reflected in patients attending MSK podiatry clinics with several sets of foot orthoses from different suppliers. It may simply be that the use of shop‐bought orthoses provide enough cushioning to improve comfort. Good patient satisfaction is reported in the management of haemarthropathy using foot orthoses in UK haemophilia cohorts.^3^, ^4^ The lack of access to MSK services reported in this study does not necessarily represent inadequate service as consultants report full access to physiotherapy and podiatry services. The clinical needs of patients with ankle and multi‐joint haemarthropathy and also understanding whether consultants make the best use of services both require further investigation.

In conclusion, all UK haemophilia centres report access to a wide range of services for foot and ankle complications, however only 40% of patients reported access to a podiatrist. This may explain why the patient's engagement with mechanical interventions such as footwear and foot orthoses was low, despite all of the patients having ankle haemarthropathy. In other diseases affecting the foot and ankle, the efficacy of footwear and orthoses in the management of pathology has been shown to improve pain and disability, whilst preventing foot deformity.^9^ In haemophilia, research is needed to understand how mechanical interventions such as footwear and foot orthoses may improve HRQoL and foot and ankle outcomes of ankle haemarthrosis and haemarthropathy. Furthermore, it is important to understand clinicians' awareness of foot and ankle interventions and the reasons why patients may not engage with foot orthoses and footwear interventions.

## Data Availability

The data that support the findings of this study are available from the corresponding author upon reasonable request.
